# The GTPase Rab37 Participates in the Control of Insulin Exocytosis

**DOI:** 10.1371/journal.pone.0068255

**Published:** 2013-06-27

**Authors:** Sanda Ljubicic, Paola Bezzi, Saska Brajkovic, Valeria Nesca, Claudiane Guay, Norihiko Ohbayashi, Mitsunori Fukuda, Amar Abderrhamani, Romano Regazzi

**Affiliations:** 1 Department of Fundamental Neurosciences, University of Lausanne, Lausanne, Switzerland; 2 EGID FR 3508, INSERM U859, Université de Lille 2, Lille, France; 3 Laboratory of Membrane Trafficking Mechanisms, Department of Developmental Biology and Neurosciences, Graduate School of Life Sciences, Tohoku University, Sendai, Japan; University of Bremen, Germany

## Abstract

Rab37 belongs to a subclass of Rab GTPases regulating exocytosis, including also Rab3a and Rab27a. Proteomic studies indicate that Rab37 is associated with insulin-containing large dense core granules of pancreatic β-cells. In agreement with these observations, we detected Rab37 in extracts of β-cell lines and human pancreatic islets and confirmed by confocal microscopy the localization of the GTPase on insulin-containing secretory granules. We found that, as is the case for Rab3a and Rab27a, reduction of Rab37 levels by RNA interference leads to impairment in glucose-induced insulin secretion and to a decrease in the number of granules in close apposition to the plasma membrane. Pull-down experiments revealed that, despite similar functional effects, Rab37 does not interact with known Rab3a or Rab27a effectors and is likely to operate through a different mechanism. Exposure of insulin-secreting cells to proinflammatory cytokines, fatty acids or oxidized low-density lipoproteins, mimicking physiopathological conditions that favor the development of diabetes, resulted in a decrease in Rab37 expression. Our data identify Rab37 as an additional component of the machinery governing exocytosis of β-cells and suggest that impaired expression of this GTPase may contribute to defective insulin release in pre-diabetic and diabetic conditions.

## Introduction

Diabetes mellitus is a metabolic disorder developing when the organism becomes unable to maintain optimal blood glucose levels [1]. Pancreatic β-cells play a fundamental role in this process. Indeed, these cells are capable of sensing circulating levels of glucose and other nutrients and to release insulin in a glucose-responsive manner [2]. In β-cells, glucose metabolism leads to the generation of ATP and to closure of ATP-sensitive K^+^-channels. This causes membrane depolarisation and Ca^2+^ entry through voltage-gated Ca^2+^-channels. The resulting increase in intracellular Ca^2+^ triggers insulin secretion [3]. Insulin exocytosis involves the formation of protein complexes that regulate each step of the secretory pathway, including docking, priming and fusion of secretory granules. Among the components orchestrating hormone release from pancreatic β-cells are the GTPases Rab3a and Rab27a that play a central role in the late events of the secretory pathway [4]. These GTPases have been found to interact with a large set of effectors and to contribute to the control of docking and fusion of insulin-containing granules. Rab37 has also been reported to participate in regulated secretion in mammalian cells. This GTPase displays high sequence homologies with Rab3a and Rab27a and has been localized on secretory granules of mastocytes [5] and Natural Killer cell secretory lysosomes [6]. Moreover, a proteomic study revealed that Rab37 is associated with insulin-containing granules in the insulinoma cell line INS-1E and in primary mice β-cells [7]. Despite these observations, the potential involvement of this GTPase in the regulation of insulin exocytosis from pancreatic β-cells has so far not been investigated.

In this study, we provide evidence that Rab37 is expressed in human islets and β-cell lines and participates in the regulation of insulin secretion. Moreover, we show that the expression of Rab37 is reduced under physiopathological conditions mimicking pre-diabetic and diabetic conditions characterized by defective insulin release.

## Materials and Methods

### Plasmids and Antibodies

EGFP-tagged wild type Rab37, Rab3a and Rab27a and their constitutively active mutants were generated as previously described [8]. Mouse polyclonal Rab37 antibody was obtained from Abcam (Cambridge, UK) while rabbit polyclonal Rab37 antibody was generated as described [8]. Mouse monoclonal anti-GFP antibody was purchased from BD Transduction Laboratories (San Jose, CA) while mouse anti-β-actin antibody was from Sigma (Saint-Louis, MO).

### Silencing of Rab GTPases and ICER

Rab37 shRNAs (shRab37 (1), shRab37 (2)) and Rab3a shRNA targeting the 19-nucleotide sequences: AGTGGTGACAGTGGATGGT, AGGGTGATCCGTTCTGAAG **,** and GGACAACATTAATGTCAAG respectively, were subcloned into the *Apa*I and *Eco*RI sites of the pSilencer1.0-U6 vector, enabling expression of short hairpin RNAs under the control of the mouse U6 promoter [8]. The Rab27a shRNA was described elsewhere [9]. A shRNA sequence directed against GFP (5′-GACGUAAACGGCCACAAGUUC-3′) was used as a negative control. For expression studies in physiopathological conditions, we used a siRNA against the repressor ICER (5′-CTGGAGATGAAACTGCTGC-3′ and 5′-CTGGAGATGAAACTGATGA-3′) [10], [11].

### Cell Culture and Transfections

The insulinoma cell lines MIN6B1 [12] and INS-1E [13], [14] were provided by Drs P. Halban (University of Geneva) and P. Maechler (University of Geneva), respectively. MIN6B1 cells were grown at 37°C and 5% CO_2_ in Dulbecco’s modified Eagle’s medium (containing 15% fetal calf serum, 70 µM β-Mercaptoethanol, 50 units/ml penicillin, 50 µg/ml streptomycin) and INS-1E cells in RPMI 1640 medium (containing 10% fetal calf serum, 70 µM β-Mercaptoethanol, 50 units/ml penicillin, 50 µg/ml streptomycin and 0.1 mM sodium pyruvate). Transient transfections were performed using Lipofectamine 2000 (Invitrogen. Carlsbad, CA). Transfection efficiency ranged between 30–50%. To mimic physiopathological conditions favoring the development of Type 1 or Type 2 diabetes, MIN6B1 cells were cultured for a) 24 h in the presence or absence of IL-1β alone (10ng/ml) or with a combination of cytokines including TNFα (10 ng/ml), IFNγ (30 ng/ml) and IL-1β (0.1 ng/ml) b) 24 h, 48 h or 72 h with 0.5 mM palmitate complexed to BSA (1∶5) c) 72 h with 2 mM cholesterol of native LDL or mildly oxidized LDL (oxLDL).

### Real-Time PCR Experiments

Total RNA was prepared using the Ambion’s RNA extraction kit (Austin, TX). Reverse transcription reactions were performed as previously described [15]. To assess shRab37 silencing efficiency or Rab37 and Rab3a expression following chronic exposure to either inflammatory cytokines, palmitate, native or oxLDL, qRT-PCR assays were carried out on a BioRad MyIQ Single-Color Real-Time PCR detection system using the BioRad IQ SYBR Green Supermix, with initial primer concentrations of 6 µM and 16 µl of RT reaction per 64 µl of PCR and an annealing temperature of 60°C. Primer sequences of Rab3a, Slp4/granuphilin and Rplp0 are those previously published [16]. Primer sequences for Rab37 were: sense, 5′-GTCTGCTTGGCTACCTCTGG-3′; antisense, 5′-GAACCCAGGTGGAAAGTTGA-3′. Normalization of each qRT-PCR reaction was performed by measuring 18S ribosomal RNA levels with the following primers: sense, 5′-GCCTTGACCTTTTCAGCAAG-3′; antisense, 5′-GGCCTCACTAAACCATCCAA-3′.

### Immunoblotting

For protein expression studies, insulinoma cell lines were placed on ice, washed twice in phosphate-buffer saline and scraped in a small volume of lysis buffer containing: 50 mM Tris-HCl, pH 7.5, 10% glycerol, 1% NP-40, 2.5 mM MgCl_2_, 80 mM KCl, 3 mM CaCl_2_, 3 mM EDTA, 1 mM PMSF, 1 µg/ml leupeptin, 2 µg/ml aprotinin, 10 mM NAF, 0.1 mM NaVO_4_ and 25 mM β-glycerol-phosphate. The cells were sonicated and centrifuged for 5 minutes at 12’000×g at 4°C. 50 µg of proteins were separated on SDS-PAGE and transferred to PVDF membranes. The membranes were incubated with specific antibodies and then with secondary antibodies coupled to horseradish peroxidase. Bound antibodies were visualized using an enhanced chemiluminescence detection system (Amersham Biosciences).

### Immunofluorescence

For immunocytochemistry, the cells were grown on polyornithine/laminin-treated glass coverslips for 3 days. Cell monolayers were rinsed twice with phosphate-buffered saline, fixed for 30 min in 4% paraformaldehyde and permeabilized for 10 min with 0.1% Saponin. Following permeabilization, the coverslips were treated with 5% BSA as a blocking solution and were then incubated overnight at 4°C either with rabbit anti-Rab37, guinea pig anti-insulin or mouse anti-Rab3a antibodies diluted in 5% BSA and 0.1% Saponin. After rinsing with phosphate-buffered saline, the cells were exposed to secondary antibodies coupled to fluorescein isothiocyanate or rhodamine for 1 h at room temperature. Coverslips were mounted on glass slides with Fluorsafe, and analyzed by confocal microscopy (Leica SP5 AOBS Confocal Microscope, Heidelberg, Germany).

### hGH Release from MIN6B1 Cells

For secretion tests, the cells were co-transfected with the hGH-encoding plasmid pXGH5 (24-well confluent dishes, 0.8 µg/well) [17]. After 4 h of incubation at 37°C, the buffer was replaced with fresh medium containing fetal calf serum, antibiotics and β-Mercaptoethanol. Three days after the transfections, the culture medium was replaced by KREBS-Ringer/bicarbonate-Hepes buffer (KRBH, pH 7.4∶127 mM NaCl, 4.7 mM KCl, 1.2 mM KH_2_PO_4_, 1.2 mM MgSO_4_, 1 mM CaCl_2_, 5 mM NaHCO_3_, 25 mM Hepes and 0.1% bovine serum albumin) containing 2 mM glucose and the cells were incubated at 37°C for 30 min. The preincubation buffer was then removed and the cells incubated in KRBH containing either 2 mM glucose (basal condition) or 20 mM glucose (stimulatory condition). The amount of hGH released into the medium and remaining in the cells under basal and stimulating conditions was quantified by ELISA (Roche Diagnostics, Rotkreuz, CH).

### Interaction of the GTPases with Putative Effectors

The interaction of Rab GTPases with their effectors was assessed by GST (Glutathione-*S*-Transferase)-pull down assays. For this purpose, MIN6B1 cells grown in 6-well confluent dishes were transfected with either pEGFP-C1 vector alone or with EGFP-tagged Rab constructs (4 µg/well). Two days later, the medium was removed and the cells washed with cold phosphate-buffer saline. They were then scraped in pull-down buffer B containing 20 mM Tris-HCl, pH 7.5, 200 mM NaCl, 10% Glycerol, 1% Triton-X-100, 5 µg/ml Aprotinin and 5 µg/ml Leupeptin. After 30 min on ice, cell lysates were centrifuged for 5 min at 4°C at 12’000×g. Lysates (500 µg) were loaded on affinity columns consisting of Rab effector domains of Rim1, Rim2, granuphilin/Slp4, Noc2, rabphilin or Slac2c/MyRIP bound to glutathion agarose beads. After 60 min at 4°C, the beads were washed three times and mixed with Laemmli buffer. At the end of the pull-down assay, the proteins retained on the affinity columns were recovered and analyzed by Western blotting.

### Total Internal Reflection Fluorescence Microscopy

For total internal reflection fluorescence (TIRF) microscopy experiments, a Zeiss Axiovert 200 inverted fluorescence microscope was modified to allow epifluorescence and evanescence field. For TIRF illuminations, the expanded beam (488 nm-568 nm argon krypton multi-line laser, 20 mW) was passed through an AOTF wavelength selector synchronized with a SNAP-HQ CCD Camera under Metafluor software control and introduced from the high numerical aperture objective lens (100 X, 1.45 NA, Zeiss, Germany). Light entering the coverslip underwent total internal reflection at the glass-cell interface (penetration depth, 90 nm). For our TIRF experiments, MIN6B1 cells were plated on glass coverslips coated with 2 mg/ml poly-L-lysine and 33.2 µg/ml laminin, and co-transfected either with shGFP or shRab37(2) and with NPY-mRFP. As a positive control, MIN6B1 cells were transfected with NPY-mRFP and plasmids encoding shRab3a or shRab27a. Two days later, NPY-mRFP-positive docked granules were imaged under 568 nm-TIRF illuminations. The cells were incubated under resting conditions, and maintained at 37°C during the whole experiment. For docking analysis, single NPY-mRFP-positive granules were counted manually and single cell area corresponding to docking surfaces (larger than 50 µm^2^) measured. To observe exocytosis of NPY-mRFP at the single-vesicle level, we used 568 nm-TIRF illumination as described [18]. Single fusion events from NPY-mRFP granules were visually recognized and counted manually. Images were acquired every 200 ms.

### Statistical Analysis

Generally data from 3–7 independent experiments were averaged. Error bars represent the standard error of the mean (SEM) or, when indicated, standard deviations (SD). The experiments were analyzed using the SAS statistical package (SAS Inc., Cary NC, USA). For multiple comparisons, variance analysis (ANOVA) was followed by the Bonferroni multiple comparisons post-hoc test with a significance limit set at p<0.05 (p<0.01, or p<0.001).

## Results

### Rab37 is Expressed in β-cells and Colocalizes with Dense-core Insulin-containing Granules

**Figure 1:**
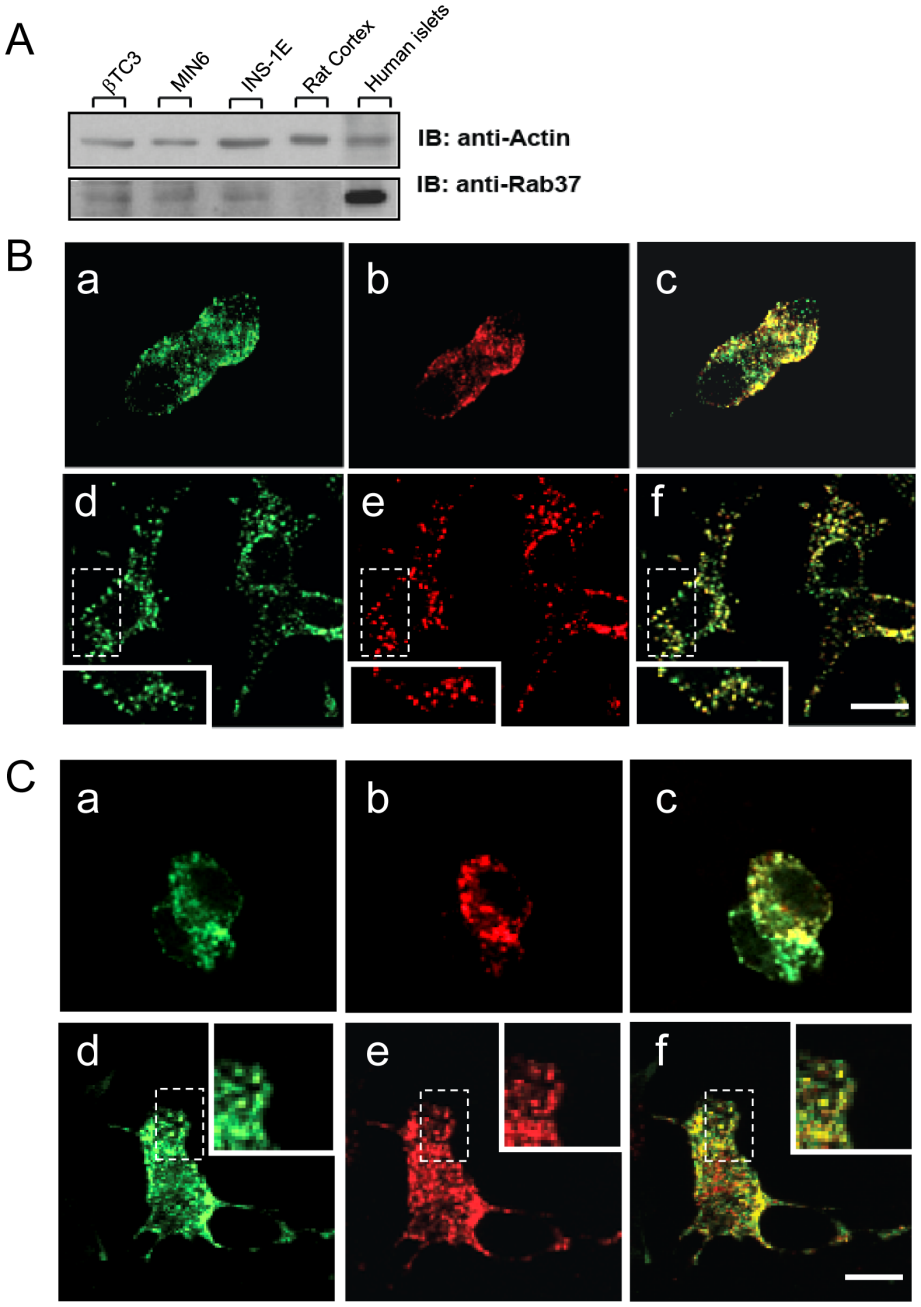
Rab37 GTPase is expressed in β-cells and is localized on insulin-containing granules. **A**. Rab37 expression in insulinoma cell lines (in βTC3, MIN6B1 and INS-1E cells), rat cortex and human pancreatic islets. Total lysates were immunoblotted with an anti-mouse Rab37 polyclonal antibody (detected band at 25 kDa). Protein loading was verified with an anti-mouse actin monoclonal antibody. **B**. Insulinoma cell lines MIN6B1 (**upper panel**) and INS-1E (**lower panel**) were stained for Rab37 (a,d) and either insulin (b) or the GTPase Rab3a (d). After immunocytochemistry, the cells were observed on a confocal microscope for colocalization (c,f). Insets represent higher magnifications of the boxed regions. **C**. MIN6B1 (**upper panel**) and INS-1E (**lower panel**) cells were co-transfected with Rab37wt-EGFP (a,d) and with NPY-mRFP (b,e). Two days later, the cells were fixed and observed on a confocal microscope. Rab37wt-EGFP co-localizes with NPY-containing large dense core granules (c,f). Insets represent higher magnifications of the boxed regions.

We first investigated the expression and subcellular localization of Rab37 in pancreatic β-cells. Western blotting analysis showed that the GTPase is expressed in several insulin-secreting cell lines, including βTC3, INS-1E and MIN6B1, and is particularly abundant in human islets **(** **Fig. 1A** **)**. In contrast, in agreement with previously published data [5], Rab37 was not detectable in extracts of rat cortex. A proteomic study revealed that Rab37 is associated with insulin-containing granules of INS-1E cells [7]. Indeed, analysis of INS-1E and MIN6B1 cells by immunolabeling demonstrated a colocalization of Rab37 with Rab3a (**Fig. 1B**) and Rab27a (**Fig. S1**), two GTPases associated with insulin-containing granules [19]. Moreover, a fluorescently labelled construct encoding wild-type Rab37 fused to EGFP (Rab37wt-EGFP) transfected in INS-1E or MIN6B1 cells colocalized with fluorescently labelled Neuropeptide Y (NPY-mRFP), which is specifically targeted to large-dense core vesicles (LDCVs) [20] (**Fig. 1C**). Taken together these observations confirm the expression of Rab37 in β-cells and its association with insulin-containing granules.

### Impact of Rab37 Knock-down on Glucose-induced Secretion

**Figure 2:**
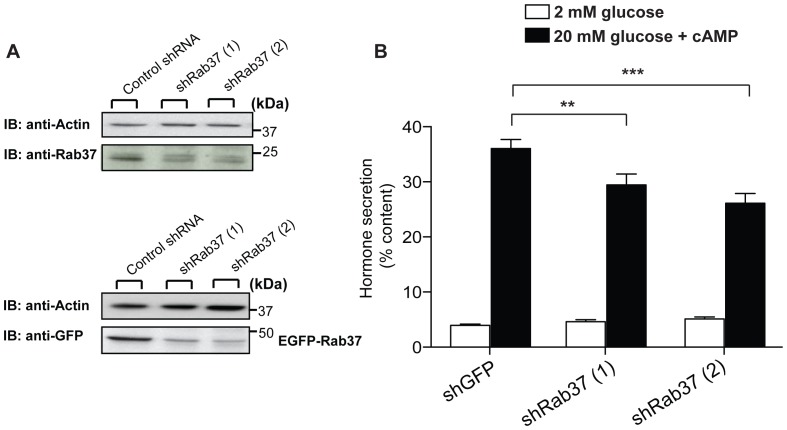
Rab37 knock-down impairs hormone secretion induced by secretagogues. **A**. The level of endogenous Rab37 (**upper panel**) and exogenously expressed Rab37-EGFP (**lower panel**) were estimated by immunoblotting in cells transfected with control shRNA, shRab37 (1) or (2). Equal loading of the lanes was verified using an antibody against actin. **B**. To assess hormone release, the cells were transiently co-transfected with a plasmid encoding the human growth hormone and with plasmids driving the expression of shRNAs against GFP (control) or with two different shRNAs against Rab37 (shRab37 (1) or (2)). Three days later, the cells were tested for hormone release. For this, they were maintained at 2 mM glucose, (open bars) and then incubated in stimulating solutions (filled bars) containing 20 mM glucose and the cAMP-raising agents Forskolin and IBMX. The amount of hGH expressed by the cells and released in the medium was quantified by ELISA. Hormone secretion under basal and stimulatory conditions is given as % of hGH cellular content. The results are the means of five independent experiments ± SEM. ** p<0.01, *** p<0.001. One-way ANOVA.

To investigate the functional role of Rab37 in pancreatic β-cells, we generated two shRNAs directed against Rab37 (shRab37 (1), shRab37 (2)). The efficiency of these constructs in decreasing Rab37 expression was assessed in MIN6B1 cells three days after transfection. Transfection efficiency under our experimental conditions was estimated to be about 40%. As shown in **Fig. S2** transfection of shRab37 (1) and (2) reduced the level of the Rab37 transcript by 50% and 65%, respectively, and decreased the expression of the protein by more than 50% (**Fig. 2A** **, top panel**). Moreover, the shRNAs strongly diminished the expression of a co-transfected EGFP-tagged Rab37 construct (**Fig. 2A** **, bottom panel**). The specificity of the shRNAs was tested by analysing their impact on the expression Rab3a and Rab27a, two closely related GTPases. The results obtained show that shRab37 plasmids are not affecting the level of exogenously or endogenously expressed Rab3a and Rab27a (**Fig.** **S3**).

Rab GTPases are important regulators of hormone and neurotransmitter release. In pancreatic β-cells, Rab3a and Rab27a play key roles in the process of insulin secretion [4]. Indeed, animals lacking either Rab3a or Rab27a are glucose intolerant and display severe insulin exocytosis defects [19], [21]. Rab37 is both structurally and evolutionarily related to these two regulatory GTPases. However, so far no information is available about a possible involvement of Rab37 in the control of insulin exocytosis. To investigate the function of Rab37 in β-cells we studied hormone secretion in cells in which the endogenous level of the GTPase is reduced by RNA interference. MIN6B1 cells were co-transfected with plasmids encoding shRNAs against either GFP (control) or Rab37 (shRab37 (1), shRab37 (2)) and with a plasmid encoding the human Growth Hormone (hGH). When expressed in β-cells, hGH is targeted to secretory granules and is co-released with insulin [22]. Therefore, hGH secretion can be used to selectively monitor the secretory process in cells expressing the shRNAs. Three days after transfection, the cells were incubated at resting glucose concentrations (2 mM glucose) or under stimulatory conditions (glucose 20 mM, Forskolin 10 µM and IBMX 0.1 mM) (**Fig. 2B** **)**. Hormone secretion at 2 mM glucose was similar in control and shRab37 transfected cells. In contrast, hormone release elicited by 20 mM glucose and cAMP-raising agents was reduced by 20% and 30%, (p<0.01) in cells expressing the shRab37 (1) and (2), respectively. Together, these observations suggest that Rab37 is required to maintain optimal secretory functions in pancreatic β-cells.

### Rab37 Knock-down Affects Docking and Exocytosis of Insulin-containing Granules

**Figure 3:**
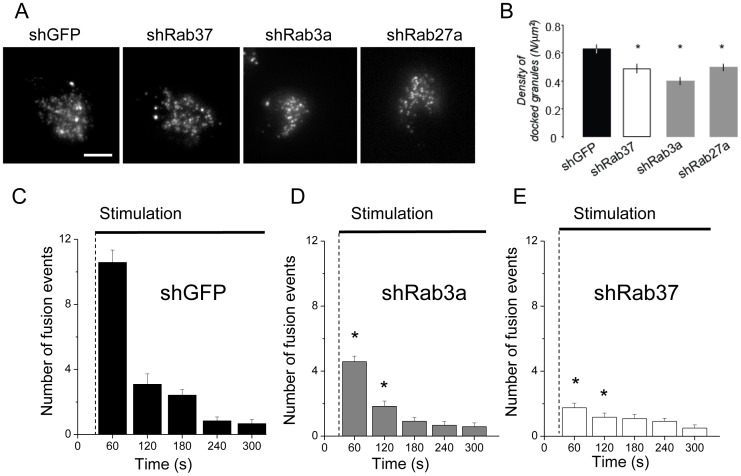
Rab37 participates in docking of insulin-containing granules. **A.** Representative TIRF images showing docked NPY-mRFP containing granules in MIN6B1 cells transfected either with control shGFP, shRBa3a, shRab27a or with shRab37 (2). **B.** Analysis of the docked granules detected by TIRF illumination in the experiments shown in A. Histograms show the quantification of the number of docked granules per surface (µm^2^). Results are means ± SEM (shGFP, n = 41 cells; shRab37 (2), n = 38 cells; shRab3a, n = 33 cells; shRab27, n = 37 cells). * p<0.05 one-way ANOVA test. **C.** Quantification of the exocytotic events from NPY-mRFP transfected MIN6B1 cells expressing control shGFP, shRab3a or shRab37 (2). Exocytosis was triggered when indicated (Stimulation) by treating the cells with glucose, Forskolin and IBMX. Histograms represent the number of fusion events counted every 60 s (n = 12). The results shown are the means ± SEM. p * <0.05 unpaired *t*-test.

The Rab GTPases Rab3a and Rab27a are critical regulators of docking as well as exocytosis of secretory vesicles in neurons and endocrine cells [8]. In β-cells, Rab3a and Rab27a regulate the last steps of insulin secretion and control the docking/tethering event [21], [23]. In view of the impact of Rab37 knock-down on hormone secretion we tested the role of the GTPase in insulin granule docking and exocytosis. For this purpose, MIN6B1 cells were analyzed by TIRF microscopy, which permits to selectively visualize fluorescently-labelled vesicles located in close proximity to the plasma membrane (less than 90 nm). The granules in close apposition to the plasma membrane were counted in MIN6B1 cells transfected with shRab37 (2), which reduced more efficiently glucose-stimulated hormone secretion, and its effect was compared with shRNAs against GFP, Rab3a or Rab27a. The granules were visualized by co-transfecting the cargo protein NPY-mRFP. Our results show that shRab37 significantly affects docking of secretory granules and reduced by more than 20% the number of secretory vesicles localized close to the plasma membrane (**Fig. 3A,B**). Interestingly, the impact of Rab37 silencing was comparable to the effect obtained in cells expressing either shRab3a or shRab27a, two constructs that have previously been reported to reduce granule docking in PC12 cells [8]. Expression of either shRab3a or shRab27a diminished the number of plasma-membrane-docked vesicles by 36% and 22% respectively (**Fig. 3A, B**). These findings are unlikely to be attributable to differences in cellular NPY-mRFP expression because the levels of the granule marker (measured as Total_NPY-mRFP_ Fluorescence/Area_cell_) in the cells transfected with shRNAs against Rab3a, Rab27a and Rab37 selected for analysis did not vary significantly.

We also explored the effect of shRab37 and shRab3a on stimulus-evoked exocytosis of insulin-containing granules. In shGFP-transfected MIN6B1 cells stimulation with a combination of secretagogues including glucose (20 mM), KCl (30 mM), Forskolin (10 µM) and IBMX (100 nM) induced an immediate increased in the frequency of exocytic events of NPY-mRFP dense core granules (∼7 fold of the basal rate in the first 120 s after the beginning of the stimulus, **Fig. 3C**). Transfection of either shRab3a or shRab37 (2) decreased significantly the frequency of the fusion events (−53% and −80%, respectively, in the first 120 s following the stimulation; **Fig. 3D, E**, n = 24 cells, p>0.05).

### Rab37 does not Interact with Rab3a and Rab27a Effector Proteins

**Figure 4:**
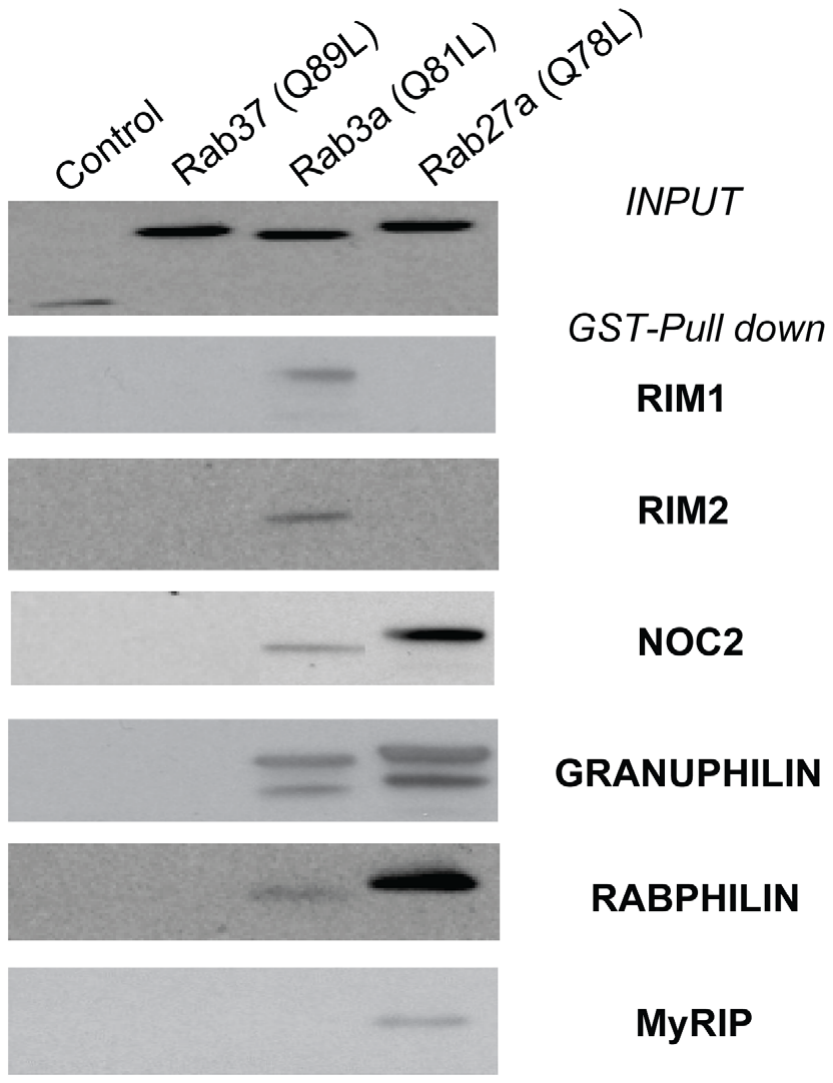
Rab37 does not interact with Rab3a and Rab27a effectors. MIN6B1 cells were transfected either with pEGFP-C1 empty vector or with the constitutively active mutants of different Rabs (Rab3a, Rab27a or Rab37). GST-fusion proteins of different potential downstream effectors (Rim1, Rim2, Noc2, granuphilin, rabphilin and Slac-2/MyRIP) were immobilized on glutathion-agarose beads. Five hundred micrograms of transfected MIN6B1 cell lysates were loaded on the gluthation-agarose beads. pEGFP-labelled Rabs remaining attached to the affinity columns were detected with an anti-GFP antibody (top panel: INPUT, 50 µg proteins, anti-GFP; lower panels: GST-Pull down, anti-GFP). Granuphilin, rabphilin and Noc2 interacted with both Rab27a and Rab3a, whereas Rim1 and Rim2 bound only to Rab3a and MyRIP to Rab27a. None of the downstream effectors tested interacted with Rab37.

Rab3 and Rab27 bind to a variety of downstream effectors [9], [19], [21], [22], [23]. To test a possible interaction of Rab37 with Rab3a and/or Rab27a downstream effectors, we performed *in vitro* GST-Pull down experiments. In agreement with previously published data [24], Rab3a and Rab27a were both able to interact with Noc2, granuphilin and Rabphilin (**Fig. 4**). In addition, Rab3a interacted with Rim1 and 2, whereas Rab27a associated with Slac-2/MyRIP. However, none of the tested effectors was able to interact with Rab37 (**Fig. 4**). We have previously shown that Calmodulin (Ca^2+^/CAM) can bind to Rab3a in a Ca^2+^-dependent manner [25] through a specific domain of the GTPase (Lys62-Arg85) including the basic amino acids Arg66-Arg70. Alignments of the Ca^2+^/CAM-Rab3a-interacting sequence Lys62-Arg85 with the corresponding amino acids of three other members of the Rab GTPases family (Rab27a, Rab37 and Rab1a) shows that the arginine at position 66 that is essential for Ca^2+^/CAM binding is not conserved in Rab37, rendering very unlikely the possibility of an interaction with this Rab3-specific effector (**Fig. S4**).

### Rab37 Expression in Physiopathological Conditions

**Figure 5:**
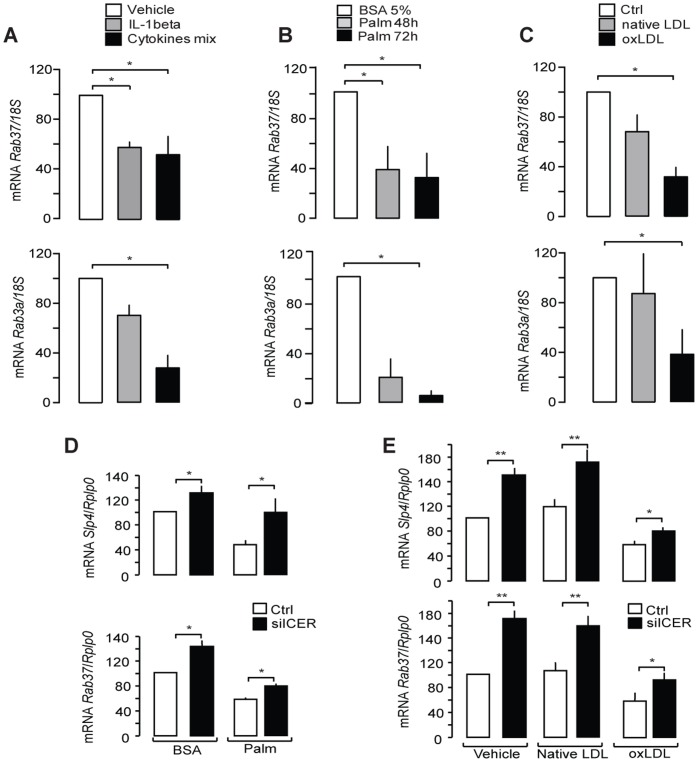
Effect of prolonged exposure to physiopathological conditions on Rab37 expression. Rab37 and Rab3a mRNA levels in MIN6B1 cells exposed for 24h to inflammatory cytokines (IL-1β alone or a mix of IL-1β TNFα, and IFNγ) (**A**), to palmitate (0.5 mM, Palm) (**B**) or for 72 h to 2 mM native or mildly oxidized LDL (oxLDL) (**C**) were quantified by qRT-PCR. MIN6B1 cells were transfected with control siRNA (siGFP, open bars) or siICER (filled bars) and cultured with either BSA or 0.5 mM palmitate for 48 h (**D**) or with vehicle, 2 mM native or mildly oxidized LDL for 72 h (**E**). The mRNA levels of Rab37 and granuphilin/Slp4 were quantified by qRT-PCR. The mRNA levels were normalized either against 18S (**A–C**) or against the housekeeping gene acidic ribosomal phosphoprotein P0 (Rplp0) (**D–E**). The expression levels in control cells were set to 100%. Data are means of ± SEM of at least 3 independent experiments (* p<0.05; ** p<0.01, one-way ANOVA).

We have previously shown that the expression of Rab3a, Rab27a and Slp4/granuphilin is altered upon exposure of β-cells to conditions mimicking those encountered in Type 2 diabetes [16], [26]. To assess whether Rab37 expression is also affected under conditions leading to β-cell failure, MIN6B1 cells were chronically exposed to proinflammatory cytokines, palmitate or oxidized Low-density Lipoproteins (oxLDL). As shown in **Fig. 5A**, Rab37 mRNA levels are reduced to the same extent as Rab3a after treatment of insulin-secreting cells with a mixture of proinflammatory cytokines including IL-1β, TNFα, and IFNγ or with high doses of IL-1β alone. Moreover, chronic exposure to either palmitate (0.5 mM) (**Fig. 5B**) or oxLDL (2 mM) (**Fig. 5C**) reduced mRNA levels of both Rab37 and Rab3a. We previously reported that the decrease in Rab3a and granuphilin expression observed in the presence of oxLDL or palmitate is due to activation of the Inducible cAMP Element Repressor (ICER) [11], [16]. We found that this transcriptional repressor is also involved in the reduction of Rab37. Indeed, silencing of ICER was able to partially restore the level of Rab37 mRNA in MIN6B1 cells chronically exposed to palmitate or oxLDL (**Fig. 5D–E**).

## Discussion

Pancreatic β-cells possess a sophisticated secretory machinery allowing them to maintain optimal insulin exocytosis. Fine-tuning of insulin release relies on the formation of SNARE complexes, the activation of Ca^2+^-binding proteins and on the function of several GTPases including the members of the Rab3 and Rab27 families [27]. Indeed, these GTPases are important components regulating the final events of insulin exocytosis, such as granule docking [28] and functional defects involving them could play a role in the development of metabolic diseases such as diabetes [27]. Recently, proteomic studies have shown that Rab37, another GTPase closely related to Rab3a and Rab27a, is highly enriched in β-cell lines and mouse pancreatic islets [7], [29]. In contrast, this GTPase is not detectable in neuroendocrine cell lines such as PC12 [8]. Rab37 has been suggested to participate in the control of exocytosis from mast cells [5]. However, no information was available on the function of this GTPase in endocrine cells. Here we confirm that Rab37 is expressed in insulin-secreting cells and human pancreatic islets and demonstrate that the GTPase co-localizes with Rab3a on insulin-containing granules. Moreover, taking advantage of an RNA interference strategy, we could demonstrate that Rab37 is involved in the process of insulin exocytosis elicited by post-prandial glucose concentrations. As is the case for Rab3a and Rab27a, Rab37 appears to be involved in the final steps of the secretory pathway. In fact, silencing of this GTPase resulted in a decrease in the number of granules in close apposition to the plasma membrane and in the number of exocytotic events in response to secretagogues, suggesting impairment in the docking and fusion processes. These observations do not exclude a contribution of Rab37 in other steps of the secretory process such as transport of insulin-containing granules that will need to be investigated in future experiments with specific technical approaches.

In view of the sequence similarities between Rab37, Rab3a and Rab27a and the analogous impacts on insulin secretion it was reasonable to expect the interaction of Rab37 with at least some of the well characterized downstream effectors of Rab3a and/or Rab27a. However, GST-pull down experiments revealed that Rab37 is unable to bind to known effector proteins. Thus, the mode of action of Rab37 appears to be distinct from that of the other GTPases controlling docking and fusion of insulin-containing granules. At present, we don’t know whether Rab37 and the other Rab GTPases associated with insulin-containing granules play redundant roles, act sequentially or regulate the fate of different granule pools [30], [31]. Images taken by confocal microscopy did not reveal major differences in the subcellular localization of Rab37 and Rab3a. However, a detailed analysis of the spatial distribution and the activation state of the different Rab GTPases associated with insulin-containing secretory granules will need to be carried out to definitively address this point.

Interestingly, we found that the expression of Rab37 is strongly reduced following exposure of insulin-secreting cells to proinflammatory cytokines, palmitate or oxLDL particles. These conditions have been reported to cause β-cell dysfunction [32], [33] and are believed to mimic physiopathological conditions that favor the development of diabetes. Our data indicate that the decrease in Rab37 expression is at least in part mediated by ICER, a transcriptional repressor that is believed to contribute to β-cell failure evoked by chronic hyperglycemia, hyperlipidemia and oxLDL [11], [16], [34]. We have previously shown that induction of ICER leads to reduced insulin expression and diminishes transcription of the genes encoding Rab3a, Rab27a and their effectors Slp4/granuphilin and Noc2 [11], [16]. Thus, activation of ICER may permit to achieve a coordinated shut down of a set of genes involved in exocytosis. This will attenuate hormone secretion under conditions of prolonged stimulation of β-cells potentially contributing to the release of insufficient insulin to cover the organism needs and to the development of diabetes.

In summary, the data obtained in the present study point to Rab37 as a novel component participating in the cascade of events controlling insulin exocytosis. The expression of this Rab GTPase is strongly impaired under conditions resulting in β-cell dysfunction. Future studies in animal models will help clarifying whether diminished expression of Rab37 contributes to defective insulin secretion in pre-diabetic and diabetic conditions.

## Supporting Information

Figure S1 **Rab37 GTPase co-localizes with Rab27a-containing granules.** Confocal images showing a MIN6B1 cell where endogenous Rab37 (red staining) co-localizes with endogenous Rab27a (green staining). Right images represent higher magnifications of the boxed regions.
(PDF)

Figure S2 **Efficiency of Rab37 silencing by RNA interference.** The silencing efficiency of Rab37 was tested by transfecting MIN6B1 cells either with a control shRNA (shGFP) or with two different shRNAs against the GTPase (shRab37 (1) or (2)). Three days following the transfection, total RNAs were extracted in each condition. Rab37 mRNA levels were measured by quantitative Real time-PCR. The results represent the means of four independent experiments ± SEM.
(PDF)

Figure S3 **Efficiency of shRab37 on exogenous and endogenous Rab3a and Rab27a.** The impact of shRab37 on exogenously expressed Rab3a and Rab27a (**A**) and on the endogenous level of these GTPases in PC12 cells (**B)** was studied by immunoblotting using anti-Rab3A mouse monoclonal antibody (dilution, 1∶250) and anti-Rab27A mouse monoclonal antibody (1∶1000 dilution). Equal protein loading of the lanes was verified using an antibody against actin. The positions of the molecular weight markers are shown on the left.
(PDF)

Figure S4 **The effector domain of Rab37 does not contain the two arginines required for Ca^2+/^calmodulin binding.** Alignments of the Ca^2+^/CAM-Rab3a-interacting amino acid sequence (Lys62-Arg85) including two positively charged amino acids (Arg66-Arg70) with the analogous sequences of Rab27a, Rab37 and Rab1a. As is the case for Rab27 and Rab1a, Arg66 is not conserved in Rab37 rendering an interaction of this GTPase with Ca^2+^/CAM very unlikely.
(PDF)
