# Correction: The GTPase Rab37 Participates in the Control of Insulin Exocytosis

**DOI:** 10.1371/annotation/27fb555c-4365-4c5f-a1c9-42b4e9608f20

**Published:** 2013-07-09

**Authors:** Sanda Ljubicic, Paola Bezzi, Saska Brajkovic, Valeria Nesca, Claudiane Guay, Norihiko Ohbayashi, Mitsunori Fukuda, Amar Abderrahmani, Romano Regazzi

The name of the eighth author was spelled incorrectly. The correct name is: Amar Abderrahmani.

