# Supplementary figures and images for: The GTPase Rab37 Participates in the Control of Insulin Exocytosis
Source: PLoS One. 2013 Jun 27;8(6):e68255. doi: 10.1371/journal.pone.0068255 (PMC3694898; doi:10.1371/journal.pone.0068255)

Supplementary Figure 1

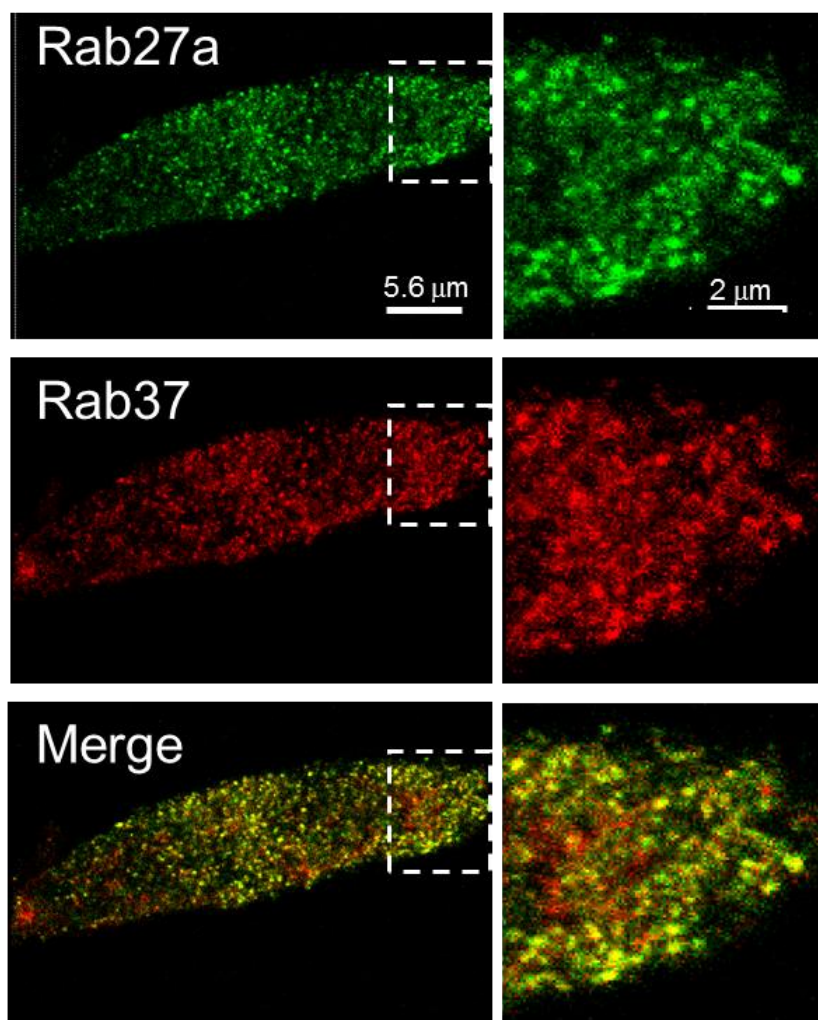

Supplement: Figure S1 — Rab37 GTPase co-localizes with Rab27a-containing granules. Confocal images showing a MIN6B1 cell where endogenous Rab37 (red staining) co-localizes with endogenous Rab27a (green staining). Right images represent higher magnifications of the boxed regions. (PDF) [file pone.0068255.s001.pdf]

Supplementary Figure 2

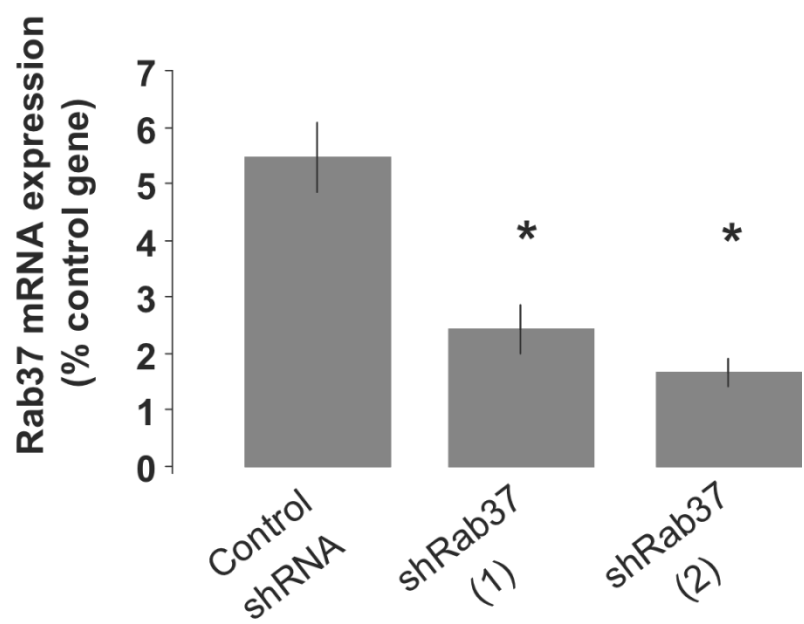

Supplement: Figure S2 — Efficiency of Rab37 silencing by RNA interference. The silencing efficiency of Rab37 was tested by transfecting MIN6B1 cells either with a control shRNA (shGFP) or with two different shRNAs against the GTPase (shRab37 (1) or (2)). Three days following the transfection, total RNAs were extracted in each condition. Rab37 mRNA levels were measured by quantitative Real time-PCR. The results represent the means of four independent experiments ± SEM. (PDF) [file pone.0068255.s002.pdf]

# Supplementary Figure 3

A

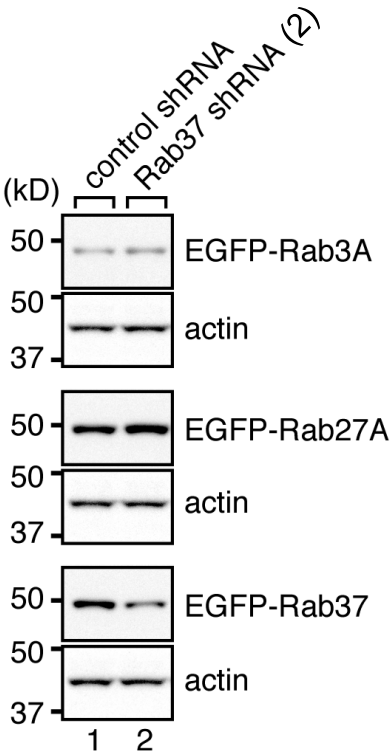

B

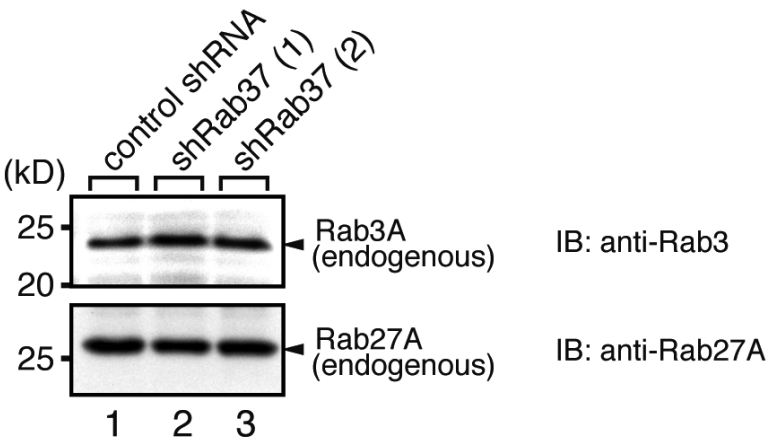

Supplement: Figure S3 — Efficiency of shRab37 on exogenous and endogenous Rab3a and Rab27a. The impact of shRab37 on exogenously expressed Rab3a and Rab27a (A) and on the endogenous level of these GTPases in PC12 cells (B) was studied by immunoblotting using anti-Rab3A mouse monoclonal antibody (dilution, 1∶250) and anti-Rab27A mouse monoclonal antibody (1∶1000 dilution). Equal protein loading of the lanes was verified using an antibody against actin. The positions of the molecular weight markers are shown on the left. (PDF) [file pone.0068255.s003.pdf]
