# Supplementary material for: The GTPase Rab37 Participates in the Control of Insulin Exocytosis
Source: PLoS One. 2013 Jun 27;8(6):e68255. doi: 10.1371/journal.pone.0068255 (PMC3694898; doi:10.1371/journal.pone.0068255)
Supplement: Figure S4 — The effector domain of Rab37 does not contain the two arginines required for Ca2+/calmodulin binding. Alignments of the Ca2+/CAM-Rab3a-interacting amino acid sequence (Lys62-Arg85) including two positively charged amino acids (Arg66-Arg70) with the analogous sequences of Rab27a, Rab37 and Rab1a. As is the case for Rab27 and Rab1a, Arg66 is not conserved in Rab37 rendering an interaction of this GTPase with Ca2+/CAM very unlikely. (PDF) [file pone.0068255.s004.pdf]

Supplementary Figure 4

		66 70		GTP-binding site	
**Rab3a**	**K**TIY	**R**NDK**R**	IKLQI	**WDTAGQE**	RY**R**
**Rab27a**	**D**GAV	**G**RGQ**R**	IHLQL	**WDTAGQE**	RF**R**
**Rab37**	**K**VVT	**V**DGA**R**	VKLQI	**WDTAGQE**	RF**R**
**Rab1a**	**R**TIE	**L**DGK**T**	IKLQI	**WDTAGQE**	RF**R**
